# Individualization of tDCS intensity according to corticospinal excitability does not improve stimulation efficacy over the primary motor cortex

**DOI:** 10.1016/j.ynirp.2021.100028

**Published:** 2021-07-08

**Authors:** Etienne Sallard, Jaimie Lee Rohrbach, Catherine Brandner, Nicolas Place, Jérôme Barral

**Affiliations:** a Institute of Psychology, Quartier Unil-Mouline, Bâtiment Géopolis, University of Lausanne, Switzerland; b Institute of Sport Sciences, Quartier Unil-Centre, Bâtiment Synathlon, University of Lausanne, Switzerland

**Keywords:** tDCS, TMS, MEP, Primary motor cortex, Individualize intensity

## Abstract

Transcranial direct current stimulation (tDCS) applied at the same intensity for an entire group of people results in wide interindividual variability, limiting stimulation efficacy. Evidence suggests that tDCS efficacy might be linked to individual corticospinal excitability (CSE) levels measured by transcranial magnetic stimulation (TMS). However, no study has attempted to individualize tDCS parameters according to the CSE level. We aimed to investigate whether the tDCS effect could be improved by individualizing stimulation intensity based on CSE measured at baseline. Fourteen participants were included in a crossover single-blinded design study where anodal (1 mA), individualized anodal (between 0.9 and 1.6 mA) and sham tDCS were applied for 14 min over the primary motor cortex. The resting motor threshold (RMT), stimulus intensity for a 1 mV response (SI1mV) and the input-output curve (I–O curve) were measured before, immediately after, 15 after and 30 min after tDCS using single pulses of TMS. The tDCS intensity in the individualized anodal condition was determined according to the RMT value at baseline (i.e., CSE level). RMT, SI1mV and I–O curve MEPs did not change after any tDCS paradigm. Our results are consistent with previous investigations that did not show an effect of tDCS on CSE and supports that tDCS protocols suffer from large interindividual variability and a lack of efficiency. This calls for further investigations to find the optimal tDCS setting to reduce the inconsistency in the results and obtain reproducible effects.

## Declarations of interest

None.

## 1 Introduction

The application of noninvasive brain stimulation, such as transcranial direct current stimulation (tDCS), enables corticospinal excitability (CSE) modulation by using a weak electrical current through the brain with surface electrodes placed on the scalp. Modulations of CSE are indexed by motor evoked potentials (MEPs) measured by electromyography (EMG) in response to single pulse transcranial magnetic stimulation (TMS). For instance, applying a 5 min period of tDCS over the primary motor cortex (M1) increases MEP amplitude, indicating a higher level of CSE (for a review see Nitsche et al., 2008; Nitsche and Paulus, 2000). Importantly, increased CSE has been related to the facilitation of neuroplasticity changes, resulting in improved motor and cognitive functions (for a review see Antal et al., 2014).

Although tDCS has become a highly popular neuromodulation technique with potential benefits in healthy and clinical populations (Brunoni et al., 2012; Dissanayaka et al., 2017), considerable inconsistencies in research outcomes cast doubt upon the reliability of this tool and limit its expected therapeutic effect (Chew et al., 2015; Horvath et al., 2015; Tremblay et al., 2016; Wiethoff et al., 2014). For instance, when anodal tDCS was applied over M1 at 1 or 2 mA, approximately half of participants did not respond to stimulation (i.e., the non-responders), as evidenced by an absence of an increase in CSE (e.g. López-Alonso et al., 2015; Wiethoff et al., 2014). Variability in the results was also demonstrated when participants showed an increase in MEP amplitude when anodal stimulation was applied at 2 mA but not at 1 mA (Ammann et al., 2017).

Multiple sources of variability have been highlighted in the inconsistent tDCS results, including brain-intrinsic (e.g., arousal, head size, cortical gyri-sulci morphologies), task-related and methodological factors (e.g. intensity, duration electrode position; Fertonani and Miniussi, 2017; Polanía et al., 2018; Ridding and Ziemann, 2010). Although the control of all these factors is a major challenge in stimulation protocols, we suggest that individually adjusted tDCS settings might overcome this variability and increase stimulation efficiency. Since the fine balance between excitation and inhibition is affected by the dose of stimulation applied through the scalp (Karrasch et al., 2004), tDCS intensity has to be adapted to each individual brain. Thus, to obtain a more efficient tDCS effect, stimulation intensity should be adapted to an individual's CSE baseline. Therefore, the individual tDCS dose-response effect needs to be understood.

Though the tDCS dose-response relation could indicate that increasing stimulation intensity might maximize CSE effects, growing evidence suggests a more complex dose-response relationship (Ammann et al., 2017; Batsikadze et al., 2013; Jamil et al., 2017; Kidgell et al., 2013; Monte-Silva et al., 2013).

The dose-response relationship has been investigated by few studies comparing different tDCS intensities and/or by splitting people into groups of high responders vs. low responders (Jamil et al., 2017; Labruna et al., 2016). In Jamil et al. (2017), tDCS was applied at different intensities (i.e., 0.5, 1, 1.5 and 2 mA) for 15 min and resulted in an increase in MEP amplitude on the abductor digiti minimi (ADM) at all intensities compared to sham tDCS. Interestingly, when the group of participants was divided according to TMS responsiveness (i.e., sensitivity to stimulation), larger CSE changes were reported i) at lower tDCS intensities (0.5 mA and 1 mA) for high responders (i.e., low TMS intensity to obtain the MEP amplitude at 1 mV), and ii) at higher intensities (1.5 mA and 2 mA) for low responders (i.e., high TMS intensity to obtain the MEP amplitude at 1 mV). Additional evidence of a relationship between tDCS efficacy and TMS responsiveness has been highlighted by a negative correlation revealing a larger increase in MEP amplitude in those who exhibited a strong response to TMS after they received 1 mA of tDCS (for similar results see also Labruna et al., 2016). Overall, these works suggest that the CSE level at baseline indexed by TMS responsiveness was likely to determine a linear relationship between tDCS intensity and CSE change. Consequently, the linear adaptation of tDCS intensity according to the CSE level might result in larger neuromodulation changes compared to nonadaptation of tDCS intensity.

This study aimed to explore individual tDCS dose responses by individualizing stimulation intensity according to the CSE level. By measuring the individual level of CSE at baseline, we could administer the most efficient tDCS dose (i.e., stimulation intensity) and provide information about how to optimize tDCS application. We predict that individualized tDCS intensity would result in larger MEP changes compared to non-individualized stimulation intensity.

## 2 Material and methods

### 2.1 Participants

Nineteen right-handed (Oldfield score: Mean ± SD: 86 ± 18) subjects were recruited from the student community of the University of Lausanne (Switzerland). Participants provided written informed consent according to the Declaration of Helsinki. The study was approved by the local research ethics committee (CER-VD 2019–00107). All participants were medication-free, had no history of psychiatric or neurologic disorders, and reported no contraindications for stimulation in the safety tDCS-TMS questionnaire (Brunoni et al., 2012). Five participants were withdrawn because of MEP artifacts. The final sample was composed of 14 participants (9 female) aged 21 ± 1 year.

### 2.2 Procedure

**Fig. 1:**
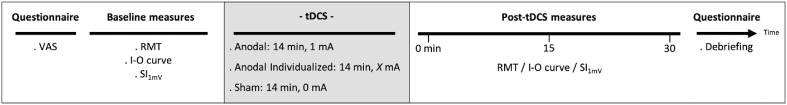
Experimental design. Representation of the timeline used in each stimulation session.

Prior to starting the experiment sessions, participants were familiarized with the stimulation procedure (TMS and tDCS). During this session (~30 min duration), stimulation was applied over the first dorsal interosseous (FDI) hot spot (i.e., the stimulation site consistently producing the highest amplitude MEPs). Once familiarization was achieved, participants performed three separate randomized experimental sessions (anodal, anodal individualized and sham conditions) lasting ~2 h with at least a 48 h wash-out period (mean ± standard error: 10 ± 6 days). Participants were blinded to the stimulation condition. All experimental sessions were conducted in the afternoon at 1 p.m. or 3:30 p.m. (experiments were conducted at the same hour for the 3 sessions for each participant). The stimulation procedure started with the identification of the FDI motor hot spot. Then, the RMT, input-output curve (I–O curve) and stimulus intensity for a 1 mV response (SI1mV) were recorded before (baseline) and after tDCS application. Post measurements were repeated three times during the 30 min following stimulation (i.e., t-0, t-15, and t-30; see the experimental design in Fig. 1). Potential adverse effects due to stimulation were reported using a debriefing questionnaire completed at the end of each experimental session.

### 2.3 Transcranial direct current stimulation

tDCS (StarStim system, Neuroelectrics Inc, Barcelona, Spain) was applied for 14 min using circular electrodes (Sponstim-25; 25 cm^2^) soaked in a saline solution (0.9 NaCl). Circular electrodes were preferred to rectangular electrodes because of a better electric field distribution (Morales-Quezada et al., 2019). Electrodes were positioned on a neoprene cap using the international 10–20 system. The active electrode was placed over the motor hotspot responsible for FDI movement, and the return electrode was placed over the right frontal eyebrow. In the anodal session, tDCS intensity was applied at 1 mA. In the sham session, tDCS ramped up and down during the first and last 30 s of the 14 min. In the anodal individualized session, intensity stimulation was adjusted according to a table based on a linear model (see Table 1). The maximal tDCS intensity (2 mA), corresponding to the higher intensity commonly used in tDCS studies (for a review see Dissanayaka et al., 2017), was related to the maximal stimulator output (MSO) of the TMS (i.e., 100% MSO). Then, according to our protocol, 100% MSO was adapted to the maximal TMS intensity used to obtain the RMT (i.e., 71% MSO); an MSO above 71% to obtain the RMT was not feasible in our study because the measurement of the I–O curves required the TMS intensity to be increased to 140% of the RMT. Thus, the maximal TMS intensity allowed was 71% MSO (e.g., for an RMT at 71% MSO, the I–O curve at 140% of the RMT was 100% MSO). The average tDCS intensity used in the anodal individualized session was 1.3 ± 0.2 mA (range: 0.9–1.6 mA).

**Table 1:** Guide to set tDCS intensity in the anodal individualized condition. The intensity of tDCS (mA) was set according to the TMS value (% MSO) obtained for the RMT.

TMS intensity (% MSO)	TMS intensity (% MSO) to obtain the RMT	tDCS intensity (mA)	tDCS density (mA/cm^2^)
98–100	70–71	2.0	0.057
93–97	66–69	1.9	0.055
88–92	63–65	1.8	0.053
83–87	59–62	1.7	0.050
78–82	56–58	1.6	0.047
73–77	52–55	1.5	0.044
68–72	49–51	1.4	0.041
63–67	45–48	1.3	0.038
58–62	41–44	1.2	0.035
53–57	38–40	1.1	0.033
48–52	34–37	1.0	0.030
43–47	31–33	0.9	0.027
38–42	27–30	0.8	0.024

### 2.4 Transcranial magnetic stimulation

TMS was delivered with Magstim 200^2^ (Magstim Co. Ltd, UK) using an eight-coil 70 mm over the left motor cortex. Single pulses were administered every 7–10 s. The intensity of TMS was set at 40% MSO and then adjusted to each participant until FDI movement could be visually observed. Once the targeted area was found, the coil position was marked on the head. The coil position (matching with the mark on the head) was systematically checked during the whole MEP measurement. CSE was measured with multiple MEP variables, such as the RMT, I–O curve and SI1mV. The RMT was determined at the lowest intensity to induce MEPs with peak-to-peak amplitudes larger than 50 μV in at least 5 out of 10 consecutive trials (Rossi et al., 2009). The I–O curves were obtained by applying TMS intensities at 120% and 140% RMT in a randomized order (10 MEPs at each intensity). An average of 10 MEPs from 100% (i.e., corresponding to the RMT) to 140% were used to represent the I–O curves. Finally, SI1mV values were obtained by adapting the TMS intensity to elicit, on average, MEPs with a peak-to-peak amplitude of 1 mV (6 MEPs). Surface EMG data were recorded with AcqKnowledge software (version 4.2) using a Biopac MP150 system (Goleta, CA, USA). Surface Ag–AgCl circular electrodes (1 cm diameter; Kendall Meditrace 100, Tyco, Canada) were attached over the belly of the FDI. The sampling frequency was determined at 2.5 kHz (gain 5000; low pass 500 Hz; high pass 1 Hz).

### 2.5 Questionnaires

At the beginning of each experimental session, the amount of sleep was reported, and the level of anxiety, mood and stress was measured using visual analog scales (VAS: 9.3 cm long) with a cross mark at the center of the line. The extremities of the lines were labeled “very relaxed” and “very anxious” for the anxiety state, “very happy” and “very unhappy” for the mood state and “very calm” and “very stressed” for the stress state. Scores were reported in percentages (i.e., 0% = absent; 100% = severe). Potential adverse effects of tDCS were reported using a debriefing questionnaire that allowed nine side effects to be rated with a score from 0 (absent) to 4 (severe; see Brunoni et al., 2011).

### 2.6 Analyses

#### 2.6.1 MEP amplitude

The peak-to-peak amplitude of MEPs for the RMT, I–O curve (RMT-100, RMT-120, and RMT-140) and SI1mV were averaged for each stimulation condition (anodal, anodal individualized, and sham) at each time (pre, t-0, t-15, and t-30) for each participant. To ensure that baseline measures were identical between stimulation conditions, repeated ANOVAs were applied at baseline (i.e., pre) for the RMT, I–O curve and SI1mV. The tDCS effects were analyzed with normalized MEP values (i.e., normalized according to the baseline). Stimulation conditions were compared using i) a 3 × 3 repeated ANOVA with the factors of stimulation (anodal, anodal individualized, and sham) and time (t-0, t-15, and t-30) for RMT and SI1mV, and ii) a 3 × 3 x 3 repeated ANOVA with the factors of stimulation, time and intensity (RMT-100%, RMT-120%, and RMT-140%) for I–O curves. Effect size (Eta squared: η2), measuring the magnitude of the difference between variables, and bayes factor (BF10), determining the strength of evidence from data about the hypotheses (i.e., alternative or null hypothesis), were reported for each MEPs analysis. Effect size is defined as small (η2 = 0.01), medium (η2 = 0.06), and large (η2 = 0.14; see Cohen, 1988)) while null hypothesis is considered as anecdotal (BF10 between 1 and 1/3), moderate (BF10 between 1/3 to 1/10) and strong (BF10 between 1/10 to 1/30; see Lee and Wagenmakers, 2013).

#### 2.6.2 Correlations

Pearson correlations were applied in each stimulation condition (anodal, anodal individualized, and sham) between the TMS intensity value (% MSO) used to obtain i) RMT and MEP values averaged across time for the RMT-100, RMT-120 and RMT-140 parameters and ii) SI1mV and MEP values averaged across time for the SI1mV parameter.

#### 2.6.3 Questionnaires

The amount of sleep was analyzed using repeated ANOVA with the factors of stimulation (anodal, anodal individualized, and sham). Scores measured on the VAS were analyzed using repeated ANOVA with the factors of stimulation (anodal, anodal individualized, and sham) and state (anxiety, stress, and mood) as within-subject factors. Responses obtained with the post-tDCS questionnaire (i.e., side-effect score) were analyzed using repeated ANOVA with the factors of stimulation (anodal, anodal individualized, and sham) and symptom (i.e. 9 symptoms; see Brunoni et al., 2011 for the list of symptoms) as within-subject factors. Effect size (η2) was also reported with the ANOVA results of the questionnaires.

## 3 Results

### 3.1 MEP amplitudes

#### 3.1.1 Baseline measures

MEP amplitudes at baseline were not significantly different between stimulation conditions for RMT (F (2; 26) = 0.07, p = 0.93, η2 = 0.004, BF10 = 0.18), SI1mV (F (2; 26) = 0.08, p = 0.93, η2 = 0.003, BF10 = 0.18) and I–O curve values (F (2; 26) = 0.03, p = 0.97, η2 = 0.000, BF10 = 5.6^−23^). A main effect of intensity was observed for the I–O curves (F (2; 26) = 82.78, p < 0.001, η2 = 0.510, BF10 = 1) with an increase in MEP amplitude according to the increase in TMS intensity. Post hoc analyses revealed differences between RMT-100 and RMT-120 (p < 0.001) and between RMT-100 and RMT-140 (p < 0.001). No interaction was observed between stimulation condition and intensity in the I–O curves (F (4; 52) = 0.20, p = 0.94, η2 = 0.001, BF = 0.08).

#### 3.1.2 Anodal, anodal individualized and sham tDCS

**Fig. 2:**
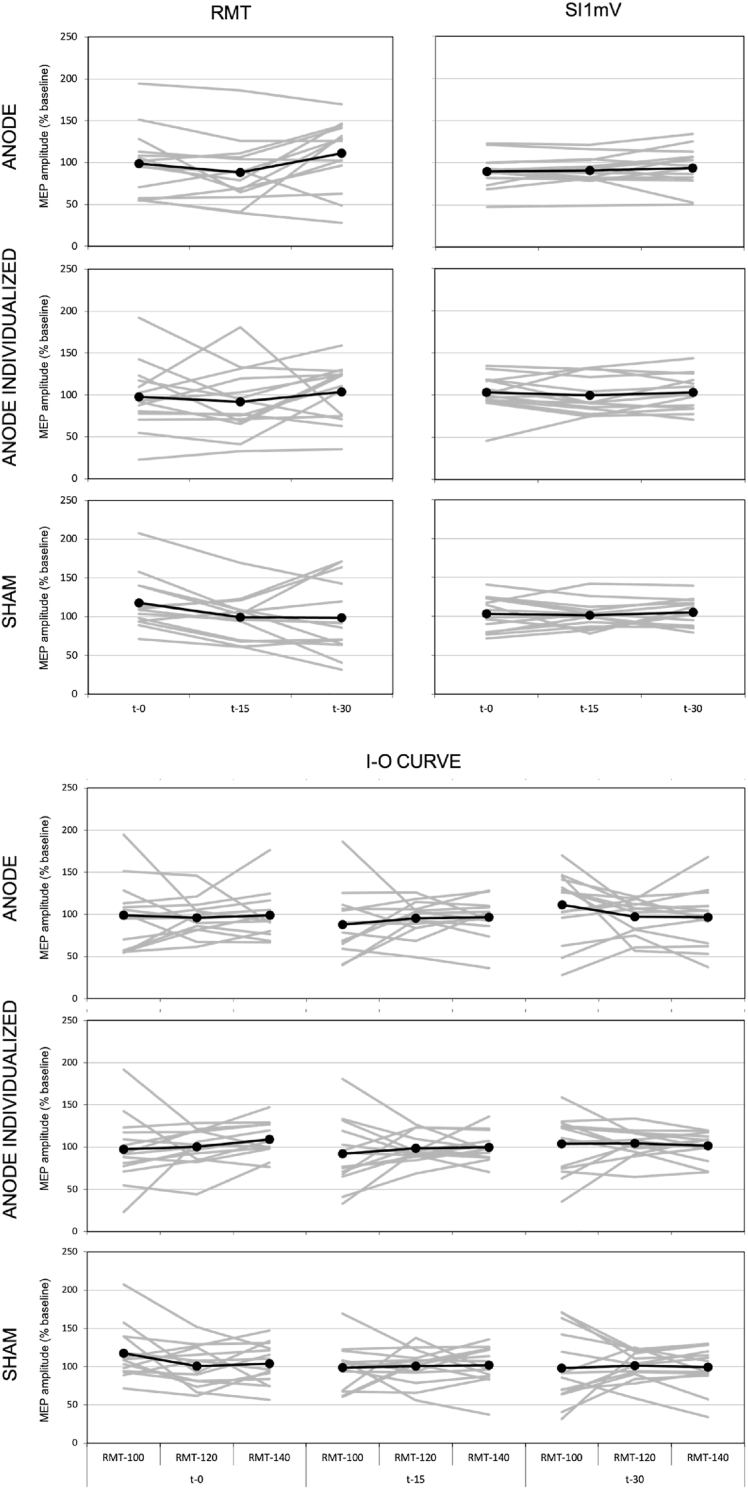
Normalized MEP amplitude in each stimulation condition at t-0, t-15 and t-30 for the RMT, SI1mV and I–O curve.

The ANOVA results revealed no CSE modulation, as evidenced by the lack of both significant main effects and interaction for the RMT, SI1mV and I–O curve measures (all p-values >0.05; see Table 2 and Fig. 2). All the bayes factors values indicate moderate or strong evidence in favor of the null-hypothesis (i.e. no difference; see Table 2).

**Table 2:** Summary of ANOVA results for RMT, SI1mV and I–O curve.

	Effects	df	F - value	P - value	η2	BF
RMT	Stimulation	2; 26	0.24	0.79	0.006	0.12
	Time	2; 26	2.8	0.08	0.02	0.32
	Stimulation x Time	4; 52	1.68	0.17	0.02	0.04
SI1mV	Stimulation	2; 26	1.75	0.19	0.07	0.13
	Time	2; 26	1.05	0.36	0.005	0.01
	Stimulation x Time	4; 52	0.19	0.94	0.002	0.1
I–O curve	Stimulation	2; 26	0.27	0.77	0.05	0.08
	Time	2; 26	2.7	0.09	0.007	0.15
	Intensity	2; 26	0.05	0.95	0.001	0.03
	Stimulation x Time	4; 52	0.86	0.5	0.005	0.01
	Stimulation x Intensity	4; 52	0.32	0.87	0.004	0.005
	Time x Intensity	4; 52	2.05	0.1	0.007	0.005
	Stimulation x Time x Intensity	8; 104	1.57	0.14	0.01	0.004

### 3.2 Pearson correlation

No significant correlation was found between TMS intensity for the RMT and time-averaged MEP amplitude for the RMT, I–O curve and SI1mV (see Table 3).

**Table 3:** Pearson correlation (r- and p-value) for each stimulation condition between the TMS intensity value (%) used to obtain the RMT and the time-averaged MEP amplitude.

TMS intensity (%MSO) to obtain RMT/time-averaged MEPs	MEP measures	r-value	p-value
Anodal	RMT-100	0.026	0.929
	RMT-120	0.093	0.751
	RMT-140	−0.111	0.706
	SI1mV	0.265	0.359
Anodal Individualized	RMT-100	0.515	0.060
	RMT-120	0.365	0.199
	RMT-140	0.045	0.879
	SI1mV	0.132	0.652
Sham	RMT-100	0.087	0.767
	RMT-120	0.326	0.256
	RMT-140	0.096	0.745
	SI1mV	0.035	0.905

### 3.3 Questionnaires

The amount of sleep was not different between stimulation conditions (F (2; 26) = 1.13, p = 0.34, η2 = 0.041). VAS scores were similar between the different states (i.e., anxiety, mood and stress) or between stimulation conditions (all p-values >0.05). Responses to the debriefing questionnaire reported at the end of each experimental session were not different between stimulation conditions (F (2; 26) = 1.46, p = 0.25, η2 = 0.006). However, scores were significantly different between symptoms (F (8; 104) = 9.76, p < 0.001, η2 = 0.238), with more tingling and sleepiness symptoms experienced regardless of the stimulation condition compared to other symptoms.

## 4 Discussion

To increase tDCS efficiency, this study attempted to adjust tDCS intensity for each individual according to the CSE level measured with TMS. Contrary to our expectation, we did not find a dose-response relationship between tDCS intensity and CSE modulation. Moreover, we did not replicate the original tDCS effect, as evidenced by the absence of CSE modulations after anodal stimulation. All these null findings are further supported by Bayesian statistics indicating a moderate or a strong evidence in favor of no difference. Below, we discuss the likely reasons for the null result.

Based on previous evidence showing a potential dose-response relationship between tDCS intensity and CSE modulations according to individual TMS responsiveness (Jamil et al., 2017; Labruna et al., 2016), we administered a personalized dose of stimulation. Our results did not demonstrate more efficient CSE modulations under individualized anodal condition compared to anodal and sham conditions, as evidenced by similar MEP amplitudes. Several reasons might explain this null result. First, the efficiency of individualized condition might be obscured by the limited range of tDCS intensity (i.e., range = 0.7 mA; from 0.9 to 1.6 mA). Larger steps between stimulation intensity (i.e., 0.5 mA steps; range = 1.5 mA; from 0.5 to 2 mA) might improve tDCS efficiency in classifying each participant more clearly according to the level of TMS responsiveness, as evidenced in Jamil et al. (2017). Second, we explored providing individualized tDCS intensity according to a linear model that did not improve stimulation efficiency. Linear models have been explored because physical models predict that current flow intensity in the brain linearly increases with applied current (Truong et al., 2013). This linear relationship has also been reported in a human model, showing that increasing the intensity of stimulation induces a larger excitability effect (Murray et al., 2015). However, other studies reported that an increased stimulation intensity did not induce greater neuromodulation (Ammann et al., 2017; Ho et al., 2016). Therefore, various linear settings and/or other rules, such as exponential or Gaussian models, must be investigated to clarify the dose-response relationship between tDCS intensity and CSE modulation.

More surprisingly, CSE after tDCS application was similar among stimulation conditions regardless of the measure (i.e., RMT, SI1mV and I–O curve) demonstrating that individualized and non-individualized anodal stimulation failed to increase CSE. Interestingly, these null effects are consistent with previous reports showing no MEP differences in RMT, SI1mV or I–O curve measures (Batsikadze et al., 2013; Horvath et al., 2015; Tremblay et al., 2016). For instance, a meta-analysis merging 6 studies applying 1 mA tDCS over M1 for a duration >7 min showed no significant RMT difference among anodal, cathodal and sham conditions (Horvath et al., 2015). Similar evidence has been reported with the SI1mV measure with no MEP modulation when comparing different intensities (i.e., 1 mA or 2 mA) and durations (i.e., 10 min or 20 min) after applying anodal tDCS (Tremblay et al., 2016). Finally, I–O curve measures also showed no CSE difference between anodal and cathodal stimulations of 2 mA applied for 20 min (Batsikadze et al., 2013). Overall, all these results demonstrate the inconsistency in tDCS effects, highlighting a wide interindividual variability that limits stimulation efficacy and reproducibility even when several sources of variability are controlled (e.g., amount of sleep, level of anxiety, mood and stress, time of the day, subjective perception of stimulations, etc.). Further investigations are needed to determine which stimulation factors are crucial to reduce interindividual variability and optimize tDCS efficacy.

It is worth noting that our results have to be interpreted with caution since the low number of participants recruited in our study might have favored our null-findings. Yet, based on a power analysis (Faul et al., 2007) for repeated ANOVA measures, a sample size of 19 participants was needed to detect a 0.8 power with an alpha error probability of α = 0.05 and an effect size of 0.25 (determined as the smallest effect size of interest for the influence of tDCS individualization; for more details see Lakens, 2013)). With a number of participants slightly below the number required, our study has a probability slightly lower than 80% to detect a true effect. The Bayesian statistics showing moderate or strong evidence in favor of the null hypothesis however support the reliability of our conclusions.

In summary, we demonstrated that individualizing tDCS intensity according to the CSE level measured by TMS responsiveness at baseline did not improve stimulation efficiency. We assume that the intensity used in the anodal individualized condition (1.3 ± 0.2 mA) might be too low to directly observe brain modulations for every participant. It is also possible that the tDCS dose-response relationship is not linear or that TMS responsiveness does not reflect tDCS responsiveness given the difficulty of setting stimulation parameters according to the CSE level. In addition, the original anodal excitatory effect was not observed, suggesting that tDCS protocols suffer from large interindividual variability and a lack of efficiency. Although it appears difficult to draw direct association between specific physical parameters and physiological effect, the understanding of the dose-response relationship in tDCS might be a major consideration to reduce inter-individual variability and obtain an efficient physiological effect for each person. In the same way that repeated TMS protocols where intensity of the stimulation is determined according to individual TMS responsiveness, a more reliable effect might be obtained in individualizing tDCS. Because stimulation intensity directly impacts the individual balance between excitation and inhibition (Karrasch et al., 2004), a specific adjustment to the cortical excitability level might reduce variability and improve tDCS efficiency. Further investigations are needed to find the optimal tDCS setting to reduce the inconsistency in the results and obtain reproducible effects. For instance, with a safety limit of tDCS reported up to 4 mA (Khadka et al., 2020; Nitsche and Bikson, 2017), individualize the stimulation using larger steps between stimulation intensity (e.g., 0.5 mA) and higher maximal tDCS intensity (i.e., up to 4 mA using specific electrodes and controller; see Khadka et al., 2020) might improve stimulation efficiency. Also, individualization of stimulation duration might improve tDCS efficiency. While anodal stimulation is generally applied between 10- and 20-min, a duration adapted to each individual might potentiate the tDCS efficiency. Thus, a time-wise monitoring with MEPs recording (e.g., every min) during tDCS administration might help to find the optimal individualized intensity and duration setting. We further encourage studies to adjust tDCS settings according to individual stimulation parameters.

## Declaration of competing interest

The authors declare that they have no known competing financial interests or personal relationships that could have appeared to influence the work reported in this paper.
